# Psychache status and associated contributing factors among the Hakka elderly in Fujian, China

**DOI:** 10.1186/s12888-024-05797-x

**Published:** 2024-05-10

**Authors:** Yating Chen, Longhua Cai, Wenqian Ruan, Lingling Zhang, Xiaojun Liu

**Affiliations:** 1 Department of Social Medicine and Health Management, School of Health Management, Fujian Medical University, Fuzhou, Fujian 350122 China; 2 Department of Epidemiology and Health Statistics, School of Public Health, Fujian Medical University, Fuzhou, Fujian 350122 China

**Keywords:** Hakka elderly, Psychache, Psychache scale (PAS), Cross-sectional study

## Abstract

**Background:**

Little is known about the state of psychological distress of the elderly in China, and research on specific subgroups such as Hakka older adults is almost lacking. This study investigates psychache and associated factors among Hakka elderly in Fujian, China.

**Methods:**

The data analysed in this study were derived from China’s Health-Related Quality of Life Survey for Older Adults 2018. The Chinese version of the Psychache Scale (PAS) was used to assess the frequency and intensity of psychache in Hakka older adults. Generalized linear regression analysis was conducted to identify the main socio-demographic factors associated with psychache overall and its frequency and intensity.

**Results:**

A total of 1,262 older adults participated, with mean scores of 18.27 ± 6.88 for total PAS, 12.50 ± 4.79 for PAS-Frequency and 5.77 ± 2.34 for PAS-Intensity. On average, females scored higher than males on PAS-Frequency (*β* = 0.84, 95% *CI* = 0.34, 1.35) and PAS-Intensity (*β* = 0.48, 95% *CI* = 0.22, 0.73). Older adults currently living in towns (*β* = -2.18, 95% *CI* = -2.81, -1.54), with their spouse only (*β* = -3.71, 95% *CI* = -4.77, -2.65), or with children (*β* = -3.24, 95% *CI* = -4.26, -2.22) were more likely to score lower on PAS-Frequency. Conversely, older adults who were regular sleepers (*β* = -1.19, 95% *CI* =-1.49, -0.88) or lived with their spouse only (*β* = -1.25, 95% *CI* = -1.78, -0.72) were more likely to score lower on PAS-Intensity.

**Conclusion:**

Among Hakka elderly, we found a higher frequency and greater intensity of psychache in females, those with poor health status, irregular sleepers, rural residents, solo dwellers, those with below CNY 10,000 in personal savings, and the medically uninsured. The study’s findings indicate that policymakers should give more attention to the susceptible population and implement practical interventions to reduce their psychological burden.

## Background

Population aging has become a significant challenge facing most countries and a global research focus for scholars [1]. Worldwide, the proportion of people aged 60 and over is growing faster than any other age group [2]. Several projections also indicate that global population aging will accelerate in the next few decades [3]. By 2050, the number of people aged 60 and over is expected to reach 22% of the world’s population [4]. The health of older people substantially influences their quality of life which can profoundly impact social development, including education, work and retirement, housing, transportation, and even built environment design. The one-child policy and increasing life expectancy in China have combined to produce the largest elderly population in any country [5]. Moreover, China’s population is one of the fastest aging in the world [6], and the health of its elderly population is becoming an urgent concern. Despite the predictability of population aging, China and the rest of the world are far from prepared to address this demographic transition [7]. For example, China’s elderly population still faces the problems of low health literacy, a high prevalence of chronic diseases, insufficient social participation, and neglect of mental health issues. Moreover, the country’s combined medical and health care service system is inadequate, medical security coordination capacity is low, and preventive medical services are limited.

The Decade of Healthy Aging (2021–2030) was approved by the World Health Assembly (WHA) in August 2020 and proclaimed by the United Nations General Assembly (UNGA) in December 2020 [8]. Healthy aging is a inclusive concept, defined by the World Health Organization (WHO) as the process of developing and maintaining the functional ability that enables well-being in older age [9]. Amid such advocacy of healthy aging, many studies have focused on older adults’ physical health [10, 11], recognizing that with rising age, the body ceases to be the trusted friend of youth and adulthood. Nevertheless, the health status of older adults undergoes gradual and complex change processes. Mental and physical illnesses coexist in many older adults [12]. With the development of modern medical technology, physical diseases are now fairly well controlled in elderly populations, but mental health is relatively more neglected. In China, psychiatric disorders such as anxiety and depression are becoming increasingly prevalent and need to be taken more seriously [13]. Conversely, some programs understand healthy aging only as emotional well-being, promoting life satisfaction [14]. Therefore, the mental health of older adults, as one key indicator of healthy aging [15], urgently requires more attention from all sectors of society.

Psychache is an unpleasant feeling (a suffering) of a psychological, non-physical origin. Psychache is the individual’s introspective experience of negative emotions such as guilt, despair, fear, and feelings of loss [16]. Anxiety, depression, physical pain, and chronic illness are common risk factors for psychache that may indicate suicide attempts [17]. Older adults die by suicide at a higher rate than any other age group in nearly every country globally [18]. Noteworthy is the fact that psychache has been particularly associated with suicide ideation and attempts [19]. The rates of suicidal thoughts and attempts are higher in patients with high psychological pain. As a socially vulnerable group, the elderly are more psychologically fragile and prone to psychological emotions such as sadness and fear or psychological disorders such as anxiety, depression, and tolerance of psychache [20, 21]. According to the WHO, over 20% of adults aged 60 and over suffer from a mental or neurological disorder [22], and a study in China indicated that about 28% of older adults reported feeling lonely [23]. The prevalence of depressive disorder and anxiety disorder in Chinese elderly over 65 years old was reported as 3.8% and 4.7%, respectively [24], entailing high economic costs and personal psychological distress. Many Chinese older adults have historically sought social support from family members. However, dramatic economic and sociocultural changes in China have left many older adults struggling to get the social support they need from their families [25], increasing their vulnerability to mental illnesses such as autism. Previous studies have shown that Chinese people are more likely to avoid or delay seeking help for mental illness than people of other countries because of their cultural environment [26]. This is why older adults should be considered a priority population.

There have been many reports on mental problems for different groups [27–29]. However, psychache falls under the category of pain science and thus differs from general psychological problems. Shneidman pointed out that psychache is the direct cause of and necessary antecedent for suicide, and that other factors such as depression and despair are only associated with suicide when linked to psychache [16]. Much of the extensive study on psychache has focused on measuring psychache [30, 31] and analyzing its relationship connects to other relevant concepts [32, 33]. In the last two decades, psychache has been widely investigated in psychiatry, especially with respect to suicide [34, 35]. Some studies have indicated that older people have the highest suicide risk of any age group [36]: among elderly individuals worldwide, the suicide rate is 31.3 per 100,000 per year, while the rate among elderly Chinese men is highest at 40.3 per 100,000 per year [37]. It is evidently necessary to study psychache in older adults. Regarding the level of psychache and related factors, Chinese and foreign scholars have mainly focused on patients suffering from certain diseases, especially cancer [38, 39], while only one study investigated older adults [40]. The “Hakka” means “guest” in Chinese, which indicates that the Hakka were historically migrant people from multiple regions. During the migration, the Hakka gradually established a village-wide living arrangement and coexisted in a large “castled house”, where they collaborated and interacted closely. However, the traditional extended family has been increasingly supplanted by private nuclear families in recent years due to lifestyle changes, and an increasing number of Hakka senior people are now living alone, which may lead to some differences existing between Hakka and other populations.Therefore, this study explores psychache in an elderly population and aims to identify its potential socio-demographic correlates using the Chinese version of the Psychache Scale (PAS). The findings will raise societal awareness about psychache in older adults and provide a reference for promoting healthy aging.

## Materials and methods

### Study design and participants

The present study is a cross-sectional, community-based survey conducted in Ninghua, Fujian. Data collection for the present study was nested in a larger cross-sectional population-based survey named the China’s Health-Related Quality of Life Survey for Older Adults 2018 (CHRQLS-OA 2018) [41]. We collected data on the socio-ecological factors and mental psychache status of the Hakka elderly. The study’s design was described in our published works [42]. A total of 1,500 questionnaires were distributed, after excluding the poorly completed questionnaires (mainly those who dropped out during of the survey), a total of 1,262 valid questionnaires were obtained, with an effective rate of 84.13%.

### Measures

Participants in this study had four dimensions of demographic characteristics: individual characteristics, behavioral lifestyle, interpersonal relationships and living/ working environment. Gender, age, self-rated health status, smoking, alcohol consumption, sleep, exercise, current residence, education level, marital status, living arrangement, personal savings (CNY), medical insurance, and work status were collected as socio-demographic data of the participants. Smoking was categorized into smoking (individuals who smoke at least one cigarette per week currently) or not smoking (individuals who have never smoked or have quit smoking), and drinking was similarly classified into the binary options of drinking (individuals who drink more than one time per week) or not drinking (individuals who never drank in the past or have quit drinking). Sleep was classified as regular if answered frequently or always have sleep regularly. Conversely, they were classified as irregular sleep. Exercise was classified as frequent if answered exercise ≥ 3 times per week and for an average of ≥ 30 min each time. Conversely, they were classified as infrequent. The measures were also reported in our published work [43].

Psychache was assessed using the Chinese version of Psychache Scale (PAS) [30, 44]. The PAS was originally developed by Holden et al. [30] in 2001, and was translated into Chinese version by Yang and Chen [44] in 2017. The total PAS is a unidimensional structure with 13 items scored on a 5-point scale, score ranged from 13 to 65, with higher scores representing heavier psychache. The PAS contains two subscales: PAS-Frequency and PAS-Intensity. PAS-Frequency included nine entries describing the frequency of pain, rated from 1 (never) to 5 (always). PAS-Intensity composed of four entries reflecting the intensity of pain, rated from 1 (strongly disagree) to 5 (strongly agree). The Chinese version PAS questionnaire was identified as having good internal consistency and reliability and can be used as a measure of psychache [30, 44, 45]. The cronbach’s α coefficient for the psychache scale in the questionnaire design was 0.97.

### Statistical analysis

The Statistical Package for the Social Sciences (SPSS) version 25.0 (SPSS/ IBM Corp, Armonk, NY, USA) was employed to perform all statistical analysis work. The test level was set at α = 0.05. A summary of the demographic characteristics of the study sample was presented in the form of frequencies and proportions. Means and standard deviations (S. D) of frequency, intensity, and total PAS were calculated by demographic characteristics to assess the distribution of psychache status among major demographic characteristics of the Hakka elderly in Fujian. To analyze the factors associated with psychache affecting older adults, the variables in each of the four levels of the social-ecological model were considered independent variables in this study. During the study, psychache scale score status was used as the dependent variable, and generalized linear regression analysis models were constructed for each level of each dependent variable separately. Upon this basis, a generalized linear regression analysis model was constructed to identify the main socio-demographic factors that best predicted the psychache of the Hakka elderly by correlating the factors at multiple levels. The coefficients (*β*) with a 95% confidence interval (95% CI) obtained from the models were reported.

## Results

### Demographic characteristic of the survey participants

The study was conducted on 1,500 subjects, but 1,262 questionnaires were completed and valid (response rate 84.13%). Among these participants, 51.43% were female, and 28.21% were between 60 and 64 years old. As shown in Table 1, more than half (66.80%) of the participants were married, 53.41% had never been educated, and 15.21% had only attended literacy classes or home school. Of the 1,262 participants, 400 respondents (31.70%) lived with their spouse only, and 435 (34.47%) lived with children. About half of the participants reported general health status (56.34%). Nearly 40% of the respondents with below CNY 10,000 in personal savings and 40.41% lived in the county. Only 8.08% were medically uninsured, the other 13.07% of respondents had urban employee medical insurance (UEBMI), and 78.85% of respondents had urban and rural resident medical insurance (URRBMI). PAS scores among different subgroups are summarized in Table 1. PAS scores were further divided into three categories: PAS-Frequency, PAS-Intensity, and total PAS. To be specific, mean scores of 18.27 ± 6.88 for total PAS, 12.50 ± 4.79 for PAS-Frequency and 5.77 ± 2.34 for PAS-Intensity.

**Table 1:** PAS mean scores among different population subgroups (*n* = 1262)

Variables	Categories	*n*	%	PAS-Frequency	PAS-Intensity	PAS
Gender	Male	613	48.57	11.63 ± 3.66	5.36 ± 1.89	17.00 ± 5.30
	Female	649	51.43	13.32 ± 5.53	6.15 ± 2.64	19.47 ± 7.92
Age (years)	60–64	356	28.21	13.63 ± 5.67	5.92 ± 2.68	19.54 ± 8.12
	65–69	248	19.65	11.55 ± 2.64	5.64 ± 1.51	17.19 ± 3.85
	70–74	227	17.99	12.12 ± 4.31	5.59 ± 1.89	17.70 ± 5.90
	75–79	204	16.16	12.04 ± 4.67	5.61 ± 2.26	17.65 ± 6.74
	≥ 80	227	17.99	12.58 ± 5.32	6.00 ± 2.90	18.58 ± 8.02
Self-rated health status	Very good/ Good	374	29.63	11.24 ± 2.51	5.15 ± 1.56	16.39 ± 3.78
	General	711	56.34	12.26 ± 4.33	5.75 ± 2.33	18.01 ± 6.42
	Very poor/ Poor	177	14.03	16.16 ± 7.62	7.13 ± 3.08	23.29 ± 10.49
Smoke	Still have	282	22.34	12.48 ± 4.79	5.60 ± 2.08	18.07 ± 6.65
	Quit/ Never smoked	980	77.66	12.51 ± 4.79	5.82 ± 2.40	18.33 ± 6.95
Drink	Still have	553	43.82	12.05 ± 4.45	5.55 ± 1.89	17.59 ± 6.09
	Quit/ Never drink	709	56.18	12.86 ± 5.01	5.94 ± 2.62	18.80 ± 7.41
Sleep	Regular	699	55.39	11.10 ± 2.55	5.05 ± 1.41	16.15 ± 3.70
	Irregular	563	44.61	14.25 ± 6.15	6.65 ± 2.89	20.90 ± 8.76
Exercise	Frequent	624	49.45	10.95 ± 2.23	5.10 ± 1.21	16.06 ± 3.14
	Infrequent	638	50.55	14.02 ± 5.99	6.42 ± 2.92	20.44 ± 8.64
Current residence	Village	478	37.88	14.65 ± 6.27	6.85 ± 3.01	21.50 ± 9.00
	Town	274	21.71	11.74 ± 3.69	5.36 ± 1.62	17.10 ± 4.99
	County	510	40.41	10.90 ± 2.33	4.97 ± 1.35	15.87 ± 3.44
Education level	Illiterate	674	53.41	13.40 ± 5.70	6.14 ± 2.73	19.54 ± 8.18
	Literacy class/ home school	192	15.21	11.52 ± 3.27	5.20 ± 1.28	16.71 ± 4.39
	Primary school	192	15.21	11.87 ± 3.09	5.42 ± 1.81	17.29 ± 4.61
	Junior high school and above	204	16.16	11.06 ± 3.11	5.40 ± 1.91	16.47 ± 4.75
Marital status	Married/ Cohabitation	843	66.80	11.73 ± 3.50	5.39 ± 1.76	17.12 ± 4.97
	Widowed	291	23.06	14.31 ± 6.39	6.68 ± 3.05	20.99 ± 9.20
	Others	128	10.14	13.51 ± 6.37	6.16 ± 3.08	19.66 ± 9.27
Living arrangement	Living alone	104	8.24	17.28 ± 8.59	7.70 ± 3.51	24.98 ± 11.98
	Living with spouse only	400	31.70	11.38 ± 2.85	5.37 ± 1.60	16.74 ± 4.12
	Living with children	435	34.47	11.97 ± 3.83	5.54 ± 2.19	17.51 ± 5.79
	Mixed habitation	235	18.62	13.44 ± 5.63	6.12 ± 2.69	19.56 ± 8.07
	Others	88	6.97	12.09 ± 3.47	5.48 ± 1.92	17.57 ± 4.94
Personal savings (CNY)	10,000 and lower	495	39.22	14.14 ± 6.29	6.45 ± 1.90	20.59 ± 8.95
	10,001 ~ 30,000	244	19.23	12.35 ± 4.35	5.61 ± 2.28	17.96 ± 6.35
	30,001 ~ 50,000	161	12.76	11.20 ± 1.83	5.45 ± 1.60	16.66 ± 3.14
	50,001 ~ 70,000	113	8.95	11.14 ± 2.21	5.35 ± 1.29	16.50 ± 3.23
	70,001 ~ 100,000	249	19.73	10.86 ± 2.19	4.96 ± 1.32	15.82 ± 3.28
Medical insurance	URRBMI	995	78.85	12.90 ± 5.09	5.96 ± 2.47	18.86 ± 7.31
	UEBMI	165	13.07	10.65 ± 1.25	4.73 ± 1.03	15.38 ± 1.90
	Uninsured/ Unknown	102	8.08	11.65 ± 4.59	5.58 ± 2.07	17.23 ± 6.48
Work Status	Occupied person	397	31.46	13.63 ± 5.61	6.19 ± 2.46	19.82 ± 7.81
	Inoccupation	865	68.54	11.99 ± 4.26	5.57 ± 2.25	17.56 ± 6.29
Total		1262	100.00	12.50 ± 4.79	5.77 ± 2.34	18.27 ± 6.88

*Note*. CNY = China Yuan; URRBMI = urban and rural resident medical insurance; UEBMI = urban employee medical insurance

### Factors affecting psychache of the Hakka elderly at each independent level

We separately included the variables in each of the four levels into the model for analysis, and the results are shown in Table 2. Among the individual trait level, differences between gender, age, self-rated health status and PAS-Frequency, PAS-Intensity, and total PAS groups were statistically significant (*P* < 0.05). Regarding the lifestyle and behavior level, the differences between smoking, sleep, and exercise and PAS-Frequency, PAS-Intensity, and total PAS groups were statistically significant (*P* < 0.05). For the interpersonal level, the differences between the subgroups of current residence, living arrangement, and PAS-Frequency were statistically significant (*P* < 0.05). The differences between the subgroups of current residence, education level, widowed, living arrangement, and PAS-Intensity were statistically significant (*P <* 0.05). The differences between the subgroups of current residence, primary education, widowed, and living arrangement and total PAS were statistically significant (*P* < 0.05). There was a statistically significant difference between personal savings, work status, PAS-Frequency, PAS-Intensity, and total PAS group at the level of life and work environment (*P* < 0.05).

**Table 2:** Generalized linear regression analysis of the PAS

Variables	Categories	PAS-Frequency			PAS-Intensity			PAS	
		β	95% CI for β		β	95% CI for β		β	95% CI for β
Gender (ref = male)	Female	1.42	(0.93, 1.90)***		0.69	(0.45, 0.94)***		2.11	(1.41, 2.82)***
Age (years) (ref = 60–64)	65 ~ 69	-2.46	(-3.18, -1.74)***		-0.42	(-0.78, -0.06)*		-2.88	(-3.92, -1.84)***
	70 ~ 74	-1.90	(-2.64, -1.16)***		-0.49	(-0.87, -0.11)*		-2.39	(-3.46, -1.31)***
	75 ~ 79	-1.88	(-2.65, -1.12)***		-0.42	(-0.80, 0.03)*		-2.30	(-3.41, -1.20)***
	≥ 80	-1.60	(-2.34, -0.85)***		-0.15	(-0.53, 0.23)		-1.75	(-2.83, -0.67)**
Self-rated health status (ref = very good/ Good)	General	1.25	(0.68, 1.81)***		0.60	(0.31, 0.88)***		1.85	(1.03, 2.67)***
	Very poor/ Poor	5.23	(4.43, 6.02)***		1.99	(1.58, 2.39)***		7.21	(6.06, 8.37)***
Smoke(ref = Quit/ Never smoked)	Still have	1.72	(1.04,2.40)***		1.07	(0.74, 1.40)***		2.79	(1.82, 3.77)***
Drink(ref = Quit/ Never drink)	Still have	0.45	(-0.08, 0.98)		0.16	(-0.10, 0.42)		0.61	(-0.14, 1.37)***
Sleep(ref = Irregular)	Regular	-2.40	(-3.01, -1.80)***		-1.46	(-1.75, -1.17)***		-3.86	(-4.73, -3.00)***
Exercise(ref = Infrequent)	Frequent	-2.20	(-2.81, -1.59)***		-0.79	(-1.09, -0.50)***		-2.99	(-3.86, -2.13)***
Current residence (ref = village)	Town	-2.91	(-3.57, -2.26)***		-1.54	(-1.86, -1.21)***		-4.45	(-5.39, -3.51)***
	County	-3.57	(-4.23, -2.90)***		-2.00	(-2.33, -1.68)***		-5.57	(-6.51, -4.62)***
Education level (ref = illiterate)	Literacy class/ home school	0.68	(-0.12, 1.47)		0.36	(-0.03, 0.75)**		1.03	(-0.10, 2.17)
	Primary school	1.27	(0.45, 2.10)**		0.71	(0.31, 1.12)**		1.98	(0.80, 3.17)**
	Junior high school and above	0.46	(-0.37, 1.29)		0.70	(0.29, 1.11)**		1.16	(-0.25, 2.35)
Marital status (ref = married/ cohabitation)	Widowed	0.59	(-0.11, 1.30)		0.61	(0.26, 0.95)**		1.20	(0.20, 2.20)*
	Others	-0.44	(-1.33, 0.45)		-0.04	(-0.48, 0.39)		-0.48	(-1.75, 0.79)
Living arrangement (ref = living alone)	Living with spouse only	-4.98	(-6.09, -3.87)***		-1.67	(-2.21, -1.12)***		-6.64	(-8.23, -5.06)***
	Living with children	-4.46	(-5.50, -3.42)***		-1.52	(-2.03, -1.01)***		-5.98	(-7.47, -4.49)***
	Mixed habitation	-3.62	(-4.64, -2.61)***		-1.32	(-1.82, -0.83)***		-4.94	(-6.39, -3.49)***
	Others	-5.24	(-6.49, -3.99)***		-2.09	(-2.71, -1.48)***		-7.33	(-9.12, -5.55)***
Personal savings (ref = 10,000 and lower)	10,001 ~ 30,000	-2.11	(-2.81, -1.41)***		-0.96	(-1.31, -0.61)***		-3.07	(-4.09, -2.06)***
	30,001 ~ 50,000	-2.87	(-3.68, -2.06)***		-0.95	(-1.36, -0.55)***		-3.82	(-5.00, -2.65)***
	50,001 ~ 70,000	-2.82	(-3.78, -1.87)***		-0.95	(-1.43, -0.48)***		-3.78	(-5.16, -2.40)***
	70,001 ~ 100,000	-3.08	(-3.93, -1.87)***		-1.31	(-1.73, -0.90)***		-4.40	(-5.61, -3.18)***
Medical insurance (ref = URRBMI)	UEBMI	-0.42	(-1.31, 0.48)		-0.50	(-0.94, -0.06)*		-0.92	(-2.20, 0.37)
	Uninsured/ Unknown	0.45	(-0.57, 1.47)		0.31	(-0.20, 0.81)		0.76	(-0.72, 2.23)***
Work Status(ref = Inoccupation)	Occupied person	1.53	(0.98, 2.09)***		0.58	(0.30, 0.85)***		2.11	(1.31, 2.91)***

*Notes*. *β* = Coefficient; Asterisks under (Matched) Controls denote significance (* *P*-value < 0.1, ** *P*-value < 0.05, *** *P*-value < 0.01)

### Factors affecting PAS-Frequency of the Hakka elderly

As shown in Table 3, females (*β* = 0.84, 95% *CI* = 0.34, 1.35) scored higher than males in PAS-Frequency. Those aged 65 and over scored lower in PAS-Frequency as compared to those aged 60–64. Older adults with very poor/ poor self-rated health (*β* = 2.50, 95% *CI* = 1.64, 3.37) scored higher in PAS-Frequency than those with very good/good self-rated health, indicating that they were more likely to suffer from psychache frequently. Those who smoked had higher PAS-Frequency scores than those who did not smoke (*β* = 1.31, 95% *CI* = 0.67, 1.96). A lower PAS-Frequency score was found in those Hakka elderly with regular sleep than those with irregular sleep (*β* = -1.62, 95% *CI* = -2.22, -1.02). Individuals who exercised regularly (*β* = -1.33, 95% *CI* = -2.00, -0.66) had a lower PAS-Frequency. In terms of interpersonal factors, those who were currently living in town (*β* = -2.18, 95% *CI* = -2.81, -1.54), county (*β* = -1.79, 95% *CI* = -2.55, -1.03) had lower PAS-Frequency scores than those who were living in the village. With “living alone” as the reference group, people in “living with spouse only” (*β* = -3.71, 95% *CI* = -4.77, -2.65), “living with children” (*β* = -3.24, 95% *CI* = -4.26, -2.22), and “mixed habitation” groups (*β* = -2.96, 95% *CI* = -3.92, -2.00) displayed a lower score of PAS-Frequency. Compared those with personal savings below CNY 10,000, those who were classified with CNY 30 001$\sim$50 000 (*β* = -1.03, 95% *CI* = -2.01, -0.05), 70 001$\sim$100 000 (*β* = -1.33, 95% *CI* = -2.55, -0.10) had a lower score of PAS-Frequency. PAS-Frequency scores were higher among the uninsured/ unknown (*β* = 1.94, 95% *CI* = 0.94, 2.94) compared to those covered by URRBMI.

**Table 3:** Generalized linear regression analysis of the PAS-Frequency

Variables	Categories	Model 1			Model 2			Model 3			Model 4	
		β	95% CI for β		β	95% CI for β		β	95% CI for β		β	95% CI for β
Gender (ref = male)	Female	1.42	(0.93, 1.90)***		0.78	(0.28, 1.29)**		0.75	(0.24, 1.25)**		0.84	(0.34, 1.35)***
Age (years) (ref = 60–64)	65 ~ 69	-2.46	(-3.18, -1.74)***		-2.22	(-2.91, -1.52)***		-2.38	(-3.07, -1.68)***		-2.47	(-3.18, -1.76)***
	70 ~ 74	-1.90	(-2.64, -1.16)***		-1.61	(-2.33, -0.89)***		-1.62	(-2.36, -0.89)***		-1.72	(-2.47, -0.96)***
	75 ~ 79	-1.88	(-2.65, -1.12)***		-1.96	(-2.70, -1.23)***		-2.16	(-2.92, -1.40)***		-2.53	(-3.37, -1.69)***
	≥ 80	-1.60	(-2.34, -0.85)***		-1.91	(-2.64, -1.19)***		-1.70	(-2.47, -0.92)***		-2.02	(-2.89, -1.15)***
Self-rated health status (ref = very good/ Good)	General	1.25	(0.68, 1.81)***		0.51	(-0.05, 1.07)		0.58	(-0.01, 1.14)*		0.58	(0.01, 1.16)
	Very poor/ Poor	5.23	(4.43, 6.02)***		3.50	(2.66, 4.33)***		2.73	(1.90, 3.56)***		2.50	(1.64, 3.37)***
Smoke(ref = Quit/ Never smoked)	Still have				1.55	(0.88, 2.23)***		1.34	(0.70, 1.99)***		1.31	(0.67, 1.96)***
Drink(ref = Quit/ Never drink)	Still have				0.30	(-0.23, 0.84)		0.56	(0.05, 1.07)*		0.61	(0.09, 1.13)
Sleep(ref = Irregular)	Regular				-1.64	(-2.24, -1.05)***		-1.55	(-2.15, -0.96)***		-1.62	(-2.22, -1.02)***
Exercise(ref = Infrequent)	Frequent				-1.87	(-2.47, -1.26)***		-1.52	(-2.17, -0.87)***		-1.33	(-2.00, -0.66)***
Current residence (ref = village)	Town							-2.25	(-2.88, -1.62)***		-2.18	(-2.81, -1.54)***
	County							-1.96	(-2.67, -1.26)***		-1.79	(-2.55, -1.03)***
Education level (ref = illiterate)	Literacy class/ home school							1.88	(1.09, 2.67)***		2.14	(1.31, 2.97)***
	Primary school							2.26	(1.41, 3.11)***		2.54	(1.63, 3.46)***
	Junior high school and above							0.87	(0.00, 1.73)		0.85	(-0.12, 1.83)
Marital status (ref = married/ cohabitation)	Widowed							0.65	(-0.06, 1.36)		0.51	(-0.21, 1.23)
	Others							-0.82	(-1.68, 0.03)		-1.09	(-1.95, -0.22)*
Living arrangement (ref = living alone)	Living with spouse only							-3.61	(-4.66, -2.55)***		-3.71	(-4.77, -2.65)***
	Living with children							-3.37	(-4.38, -2.36)***		-3.24	(-4.26, -2.22)***
	Mixed habitation							-3.04	(-3.99, -2.08)***		-2.96	(-3.92, -2.00)***
	Others							-4.58	(-5.77, -3.39)***		-4.77	(-5.93, -3.56)
Personal savings (ref = 10,000 and lower)	10,001 ~ 30,000										-0.54	(-1.24, 0.16)
	30,001 ~ 50,000										-1.03	(-2.01, -0.05)*
	50,001 ~ 70,000										-0.91	(-2.15, 0.32)
	70,001 ~ 100,000										-1.33	(-2.55,-0.10)*
Medical insurance (ref = URRBMI)	UEBMI										0.08	(-0.73, 0.89)
	Uninsured/ Unknown										1.94	(0.94, 2.94)***
Work Status(ref = Inoccupation)	Occupied person										0.09	(-0.51, 0.69)

*Notes*. *β* = Coefficient; Asterisks under (Matched) Controls denote significance (* *P*-value < 0.1, ** *P*-value < 0.05, *** *P*-value < 0.01)

### Factors affecting PAS-Intensity of the Hakka elderly

As demonstrated in Table 4, females (*β* = 0.48, 95% *CI* = 0.22, 0.73) scored higher than males in PAS-Intensity. Those with fair self-rated health (*β* = 0.36, 95% *CI* = 0.07, 0.65) and poor self-related health status (*β* = 0.86, 95% *CI* = 0.43, 1.30) scored higher in PAS-Intensity. Regular sleepers (*β* = -1.19, 95% *CI*=-1.49, -0.88) scored lower in PAS-Intensity than irregular sleepers. Those living in rural areas scored higher in PAS-Intensity than those living in towns or cities. PAS-Intensity score was higher among the older with “literacy class/ home school” education level (*β* = 0.82, 95% *CI* = 0.41, 1.24), and “primary school school” education level (*β* = 1.26, 95% *CI* = 0.81, 1.72) than the illiterate elderly. The scores of PAS-Intensity for the elderly with living arrangements of “living with spouse only” (*β* = -1.25, 95% *CI* = -1.78, -0.72), “living with children” (*β* = -1.12, 95% *CI* = -1.63, -0.61), “mixed habitation” (*β* = -1.06, 95% *CI* = -1.54, -0.58), and “others” (*β* = -1.85, 95% *CI* = -2.45, -1.26) were lower than those living alone.

**Table 4:** Generalized linear regression analysis of the PAS-Intensity

Variables	Categories	Model 1			Model 2			Model 3			Model 4	
		β	95% CI for β		β	95% CI for β		β	95% CI for β		β	95% CI for β
Gender (ref = male)	Female	0.69	(0.45, 0.94)***		0.32	(0.07, 0.58)*		0.47	(0.22, 0.72)***		0.48	(0.22, 0.73)***
Age (years) (ref = 60–64)	65 ~ 69	-0.42	(-0.78, -0.06)*		-0.25	(-0.60, 0.09)		-0.10	(-0.45, 0.24)		-0.17	(-0.53, 0.18)
	70 ~ 74	-0.49	(-0.87, -0.11)*		-0.36	(-0.72, 0.01)		-0.32	(-0.70, 0.05)		-0.37	(-0.76, -0.01)
	75 ~ 79	-0.42	(-0.80, 0.03)*		-0.47	(-0.84, -0.10)*		-0.26	(-0.64, 0.12)		-0.42	(-0.84, 0.00)
	≥ 80	-0.15	(-0.53, 0.23)		-0.23	(-0.59, 0.14)		0.21	(-0.18, 0.59)		0.12	(-0.31, 0.56)
Self-rated health status (ref = very good/ Good)	General	0.60	(0.31, 0.88)***		0.24	(-0.04, 0.52)		0.31	(0.03, 0.60)***		0.36	(0.07, 0.65)***
	Very poor/ Poor	1.99	(1.58, 2.39)***		1.12	(0.70, 1.54)***		0.82	(0.41, 1.24)***		0.86	(0.43, 1.30)***
Smoke (ref = Quit/ Never smoked)	Still have				0.99	(0.65, 1.33)***		0.93	(0.61, 1.26)		0.89	(0.56, 1.21)
Drink (ref = Quit/ Never drink)	Still have				0.08	(-0.19, 0.34)		0.15	(-0.10, 0.41)		0.22	(-0.04, 0.48)
Sleep(ref = Irregular)	Regular				-1.26	(-1.56, -0.96)***		-1.16	(-1.46, -0.87)*		-1.19	(-1.49, -0.88)*
Exercise (ref = Infrequent)	Frequent				-0.61	(-0.91, -0.30)***		-0.34	(-0.69, -0.02)		-0.32	(-0.67, -0.01)
Current residence (ref = village)	Town							-1.49	(-1.81, -1.18)***		-1.48	(-1.80, -1.16)***
	County							-1.61	(-1.96, -1.26)***		-1.63	(-2.01, -1.25)***
Education level (ref = illiterate)	Literacy class/ home school							0.88	(0.48, 1.27)***		0.82	(0.41, 1.24)***
	Primary school							1.30	(0.88, 1.72)***		1.26	(0.81, 1.72)***
	Junior high school and above							1.28	(0.85, 1.71)***		1.10	(0.61, 1.59)
Marital status (ref = married/ cohabitation)	Widowed							0.41	(0.06, 0.77)*		0.34	(-0.02, 0.70)
	Others							-0.28	(-0.71, 0.15)		-0.38	(-0.81, 0.05)
Living arrangement (ref = living alone)	Living with spouse only							-1.15	(-1.68, -0.62)***		-1.25	(-1.78, -0.72)***
	Living with children							-1.11	(-1.61, -0.60)***		-1.12	(-1.63, -0.61)***
	Mixed habitation							-1.07	(-1.55, -0.59)***		-1.06	(-1.54, -0.58)***
	Others							-1.81	(-2.40, -1.21)***		-1.85	(-2.45, -1.26)***
Personal savings (ref = 10,000 and lower)	10,001 ~ 30,000										-0.11	(-0.47, 0.24)
	30,001 ~ 50,000										0.11	(-0.38, 0.60)
	50,001 ~ 70,000										0.19	(-0.42, 0.81)
	70,001 ~ 100,000										-0.08	(-0.70, 0.53)
Medical insurance (ref = URRBMI)	UEBMI										-0.18	(-0.59, 0.23)
	Uninsured/ Unknown										0.83	(0.32, 1.33)**
Work Status(ref = Inoccupation)	Occupied person										-0.05	(-0.35, 0.25)

*Notes*. *β* = Coefficient; Asterisks under (Matched) Controls denote significance (* *P*-value < 0.1, ** *P*-value < 0.05, *** *P*-value < 0.01)

### Factors affecting total PAS of the Hakka elderly

Table 5 shows that females (*β* = 1.32, 95% *CI* = 0.59, 2.05) scored higher than males in total PAS. Those aged 65–69 (*β* = -2.64, 95% *CI* = -3.67, -1.62), 70–74 (*β* = -2.09, 95% *CI* = -3.17, -1.00), 75–79 (*β* = -2.95, 95% *CI* = -4.16, -1.74) and ≥ 80 years (*β* = -1.90, 95% *CI* = -3.14, -0.65) scored lower than those aged 60–64 years in total PAS. Older adults with good self-rated health scored lower than those with general (*β* = 0.94, 95% *CI* = 0.11, 1.77) and poor (*β* = 3.37, 95% *CI* = 2.12, 4.61) self-rated health in total PAS. Cigarette smokers (*β* = 2.20, 95% *CI* = 1.27, 3.13) and alcohol drinkers (*β* = 0.83, 95% *CI* = 0.09, 1.57) scored higher than non-smokers and non-drinkers in total PAS. A comparison with irregular sleepers reveals that regular sleepers (*β* = -2.81, 95% *CI* = -3.65, -1.94) scored lower on total PAS. In comparison with infrequent exercisers, it was found that frequent exercisers (*β* = -1.67, 95% *CI* = -2.63, -0.70) scored lower in total PAS. Older people living in towns (*β* = -3.42, 95% *CI* = -4.51, -2.33) or counties (*β* = -3.65, 95% *CI* = -4.57, 2.74) scored lower than those living in villages in total PAS. Higher total PAS scores were found among people with literacy class/ home school education (*β* = 2.96, 95% *CI* = 1.77, 4.16), primary school education (*β* = 3.81, 95% *CI* = 2.49, 5.12), and junior high school education and above (*β* = 1.95, 95% *CI* = 0.55, 3.36) compared to illiterate people. Those categorized as “other marital status” (*β* = -1.47, 95% *CI* = -2.71, -0.23) scored lower than in marriage/ cohabitation in total PAS. Compared to those who lived alone, those who lived with spouse only (*β* = -4.97, 95% *CI* = -6.49, -3.44), lived with children (*β* = -4.36, 95% *CI* = -5.82, -2.90), mixed living (*β* = -4.02, 95% *CI* = -5.40, -2.64), and other living arrangements (*β* = -6.60, 95% *CI* = -8.31, -4.89) were negatively associated with a higher total PAS.

**Table 5:** Generalized linear regression analysis of the total PAS

Variables	Categories	Model 1			Model 2			Model 3			Model 4	
		β	95% CI for β		β	95% CI for β		β	95% CI for β		β	95% CI for β
Gender (ref = male)	Female	2.11	(1.41, 2.82)***		1.11	(0.38, 1.83)**		1.21	(0.49, 1.93)**		1.32	(0.59, 2.05)***
Age (years) (ref = 60–64)	65 ~ 69	-2.88	(-3.92, -1.84)***		-2.47	(-3.49, -1.37)***		-2.48	(-3.47, -1.48)***		-2.64	(-3.67, -1.62)***
	70 ~ 74	-2.39	(-3.46, -1.31)***		-1.96	(-3.01, -0.92)***		-1.94	(-2.99, -0.89)***		-2.09	(-3.17, -1.00)***
	75 ~ 79	-2.30	(-3.41, -1.20)***		-2.43	(-3.39, -1.24)***		-2.42	(-3.51, -1.32)***		-2.95	(-4.16, -1.74)***
	≥ 80	-1.75	(-2.83, -0.67)**		-2.14	(-3.11, -1.01)***		-1.49	(-2.60, -0.38)**		-1.90	(-3.14, -0.65)**
Self-rated health status (ref = Very good/ Good)	General	1.85	(1.03, 2.67)***		0.75	(-0.06, 1.56)		0.89	(0.08, 1.70)*		0.94	(0.11, 1.77)**
	Very poor/ Poor	7.21	(6.06, 8.37)***		4.61	(3.40, 5.82)***		3.56	(2.36, 4.75)***		3.37	(2.12, 4.61)***
Smoke(ref = Quit/ Never smoked)	Still have				2.55	(1.57, 3.52)***		2.28	(1.35, 3.21)***		2.20	(1.27, 3.13)***
Drink(ref = Quit/ Never drink)	Still have				0.38	(-0.39, 1.15)		0.72	(-0.02, 1.45)		0.83	(0.09, 1.57)*
Sleep(ref = Irregular)	Regular				-2.90	(-3.77, -2.04)***		-2.71	(-3.58, -1.86)***		-2.81	(-3.65, -1.94)***
Exercise(ref = Infrequent)	Frequent				-2.47	(-3.35, -1.60)***		-1.86	(-2.79, -0.93)***		-1.67	(-2.63, -0.70)**
Current residence (ref = village)	Town							-3.75	(-4.66, -2.84)***		-3.42	(-4.51, -2.33)***
	County							-3.58	(-4.59, -2.56)***		-3.65	(-4.57, -2.74)***
Education level (ref = illiterate)	Literacy class/ home school							2.76	(1.62, 3.89)***		2.96	(1.77, 4.16)***
	Primary school							3.56	(2.35, 4.78)***		3.81	(2.49, 5.12)***
	Junior high school and above							2.14	(0.90, 3.38)**		1.95	(0.55, 3.36)**
Marital status (ref = married/ cohabitation)	Widowed							1.06	(0.05, 2.08)*		0.84	(-0.19, 1.88)
	Others							-1.11	(-2.34, 0.12)		-1.47	(-2.71, -0.23)*
Living arrangement (ref = living alone)	Living with spouse only							-4.76	(-6.28, -3.24)***		-4.97	(-6.49, -3.44)***
	Living with children							-4.47	(-5.92, -3.02)***		-4.36	(-5.82, -2.90)***
	Mixed habitation							-4.11	(-5.48, -2.73)***		-4.02	(-5.40, -2.64)***
	Others							-6.39	(-8.10, -4.69)***		-6.60	(-8.31, -4.89)***
Personal savings (ref = 10,000 and lower)	10,001 ~ 30,000										-0.66	(-1.67, 0.35)
	30,001 ~ 50,000										-0.92	(-2.32, 0.49)
	50,001 ~ 70,000										-0.72	(-2.49, 1.06)
	70,001 ~ 100,000										-1.41	(-3.17, 0.36)
Medical insurance (ref = URRBMI)	UEBMI										-0.10	(-1.27, 1.07)
	Uninsured/ Unknown										2.76	(1.32, 4.20)***
Work Status(ref = Inoccupation)	Occupied person										0.04	(-0.83, 0.93)

*Notes*. *β* = Coefficient; Asterisks under (Matched) Controls denote significance (* *P*-value < 0.1, ** *P*-value < 0.05, *** *P*-value < 0.01)

## Discussion

The majority of psychache research has been conducted on its correlation with mental disorders, including schizophrenia [46], bipolar disorder [47], generalized anxiety disorder [48], and obsessive-compulsive disorder [49]. There is a dearth of studies on psychache among specific subgroups such as Hakka older adults in China. Old age is a period associated with many forms of loss. A culmination of chronic physical illnesses and worsening sensory impairment can considerably affect an individual’s quality of life and psychological well-being. Previous studies have shown that the mental health of older adults is affected by gender, marital status, and social support [50]. We found that age, gender, smoking condition, sleep, exercise, current residence, living arrangement, marital status, education level and personal savings were the associated factors of psychache among Hakka elderly.

In this study of Hakka elderly, we found that females were more likely than males to feel psychache, which is consistent with prior findings [51]. The demands of traditional Chinese culture have contributed to an introversion in Chinese women’s personalities, resulting in a tendency to allow the pressure they suffer to accumulate and cause psychological pain. Chinese women are generally required to take responsibility for domestic and social work and, therefore, face home and workplace pressures. Women also experience more psychological stress than men from changes to social roles, which can easily disrupt their psychological balance. Thus, it is meaningful to support females to promptly release pent-up tension and reach out to psychological counselors for assistance if necessary.

Among the study’s participants, psychache frequency was highest among those aged 60–64 years, which seems inconsistent with previous findings [52]. Evidence suggests that the prevalence of depression tends to increase with age [53]. However some studies point to a decline in depression rates between the ages of 60 and 70, indicating that retirement is good for mental health [54]. The average age of the Fujian Hakka elderly selected for this study was higher than of the elderly in general studies, and the 60–64 age group was the youngest; members of this group may not have been fully retired yet already facing demands to care for grandchildren, resulting in more frequent psychological stress. Self-rated health status can also play a role in the psychological distress of older adults [55]. Perceiving impaired health status and cognitive decline often triggers their anxiety and, in severe cases, psychological suffering. The youth population in families are reminded to be mindful of the mental health of elderly individuals who look after their grandchildren and of parents who are in poor health conditions.

Previous studies have found that people with mental illness are more susceptible to smoking [56]. There is also some empirical evidence, mainly from the United States, that smokers with psychiatric issues make exactly as many attempts to stop as the normal smoker, but their success rate is lower [57]. Our study revealed a higher frequency of psychache among Hakka older adults who smoked, possibly because those with an indication of a mental health problem were more highly dependent on smoking and accustomed to soothing their emotions in this way [58]. Besides vulnerability to smoking, recent studies suggest that people who rely on alcohol to cope with stressful events may quickly become psychologically dependent on alcohol, and need it to withstand the resulting negative emotions from stressful events [59]. Research also suggests that heavy drinking is associated with higher levels of negative affect [60], and that older adults who experience psychological distress tend to use alcohol to enhance their positive emotions [61]. However, this study found no correlation between alcohol consumption and psychache, which may be explained by the local Chinese culture, especially the belief that “drink wine to drown your sorrow, it will heavier grow.” Social media needs to clarify awareness about the risks associated with drinking and smoking and encourage healthier ways to relieve psychological pain.

Irregular sleep and poor sleep quality are common in the elderly population. Poor sleep significantly reduces quality of life and promotes other adverse pathological outcomes, such as increased fall risk, memory problems, chronic fatigue, and weakness [62, 63]. Psychological distress has consistently been found to correlate with sleep troubles [64, 65], and this study demonstrated that psychache was more intense and/or frequent among Hakka elderly with irregular sleep. The positive association between physical exercise and mental health has also been widely discussed and extensively documented [66–68]. Older adults who engage in high levels of physical exercise tend to be more resistant to functional decline and psychological distress [68]. Therefore, we unsurprisingly found more frequent psychache in older adults who do not engage regularly in physical exercise. To avoid being psychache, elderly people should maintain regular physical activity and sleep routines.

A previous study showed that have shown that urban residents are less likely to experience depressive symptoms than rural residents [69], whereas Sengupta P et al. suggested that rural residents have a stronger sense of community belonging and are at lower risk of mental illness [70]. A study of older people in China suggested that the level of psychological pain among rural older people was more serious than among urban older [40]. Our study found that older people living in rural (vs. urban) areas were more likely to feel psychache. This could be explained by the migration of young people from rural to urban areas, resulting in a lack of social support for rural older people. Seniors living alone commonly face problems, such as loneliness, neglect, and boredom, which can affect their mental health. Previous studies show that older adults without emotional support from family members have a higher risk of psychological illness [40, 71]. Likewise, loneliness and unspoken physical and mental problems increase the frequency of psychache in our study. The government should improve welfare policies for the elderly in rural areas.

Among older adults, marital partners become increasingly important in maintaining a sense of social connectedness when family, friends, and neighbors are lost through death and relocation [72]. Marriage protects against loneliness, but not all marriages are equally protective. A study used dyadic data from the second wave (2010–11) of the National Social Life, Health, and Aging Project (NSHAP) shows that more than half of married older adults live in marital conflict, indifference, or avoidance; about one in six older married couples report moderate or high levels of loneliness [73]. Our study found a higher frequency of psychache among married/ cohabiting Hakka elders than unmarried/ divorced/ separated people. This could be explained by the older age of participants compared to other studies, since social and health transitions can increase stress and reduce marriage quality, opening the door to feelings of disconnection, isolation, and loneliness. Our findings highlight the need for further research to identify strategies to help older adults optimize their marital relationships.

Interestingly, this study found that older adults with private/ elementary school education or junior high school education had higher levels of psychache than those who were illiterate. This finding seems at odds with current understanding that low educational attainment is a risk factor for depression. Illiteracy in elderly individuals was found to be associated with a higher rate and increased severity of depression [74], while another study reported that higher educational attainment was associated with healthier psychological well-being [75]. The discrepancy in our findings may be linked to the uneven distribution of educational attainment among survey respondents, more than half of whom were illiterate. Income partly reflects an individual’s social status [76], and lower income can lead to greater financial dependence on family members, leaving older adults insecure and more prone to psychological pain, consistent with our survey. In addition, this study found that medically uninsured older adults had more intense and/or frequent psychache than those enrolled in urban employee health insurance, possibly because being uninsured means less security and more health risks [77], which can easily lead to poor mental health. Thus, the government should increase the amount of security subsidies for the elderly. It is also essential to appeal to children to visit older people frequently, providing a sense of security.

### Limitation

As the first study targeting the Hakka elderly to reveal their psychache status and associated factors, this study has several limitations. First of all, the cross-sectional design of our study limited our ability to examine causal relationships and instead only examined correlations; hence, more prospective researches are required. Secondly, psychache may indicate suicide attempts. Yet, we did not track suicide because participant information related to suicide was not the main focus of this study. Thirdly, the Hakka elderly in the present study were from Fujian, China. Nonetheless, the Hakka population is also dispersed throughout Southeast Asia, including Thailand, Malaysia, and Singapore, as well as Guangdong and Jiangxi in China. Our samples are not representative of the entire Hakka population. Therefore, the conclusions of this study need to be cautious when extrapolating. Additional research on comparing the Hakka elderly with the general Chinese elderly population is imperative. In addition, some variables were limited by the questionnaire and relied on the self-reporting for categorization are vulnerable to recall bias.

## Conclusion

In short, this study provides comprehensive understanding of psychache in the Hakka elderly of Fujian. The results revealed more intense and/or frequent psychache in females, 60–64-year-olds, those with poor self-rated health, smokers, irregular sleepers, infrequent exercisers, rural residents, solo dwellers, those with personal savings below CNY 10,000, and the medically uninsured. Hence, Hakka older adults with any of these characteristics should be given more attention. China’s policymakers and health professionals should initiate practicable interventions to reduce the psychological burden on older adults, which could be conducive to preventing and controlling their mental health problems. Future studies should conduct longitudinal investigations of the influencing factors of psychache. Studying Hakka people in different regions would produce more generalizable findings.

## Data Availability

The datasets used and/or analysed during the current study are available from the first and/ or corresponding author(s) on reasonable request because the datasets contain sensitive material that can identify participant information.

## Abbreviations

CI: Confidence interval
OR: Odds ratios
SPSS: Statistical Package for the Social Sciences
CNY: China Yuan
URRBMI: Urban and rural residents’ basic medical insurance
UEBMI: Urban employees basic medical insurance
CHRQLS-OA 2018: China’s Health-Related Quality of Life Survey for Older Adults 2018
*χ*^*2*^: Chi-square value
*β*: Coefficient
n: Subsample
